# Effect of community-led total sanitation on open defecation in Uganda: A propensity score-matched analysis

**DOI:** 10.1371/journal.pone.0329307

**Published:** 2025-07-24

**Authors:** John Bosco Asiimwe, Hellen Namawejje, Faith Rachel Mirembe, Annet Adong, Jolly Achola, Herbert Nabaasa, Jebena Mulusew, Jonathan Izudi, Damazo T. Kadengye

**Affiliations:** 1 Department of Planning and Applied Statistics, Makerere University, Kampala, Uganda; 2 Department of Statistical Methods and Actuarial Science, Makerere University, Kampala, Uganda; 3 Department of Environment Health, Ministry of Health, Kampala, Uganda; 4 Centre for Population and Applied Statistics, Makerere University, Kampala, Uganda; 5 African Population and Health Research Center (APHRC), APHRC Campus, Nairobi, Kenya; 6 Department of Community Health, Faculty of Medicine, Mbarara University of Science and Technology, Mbarara, Uganda; 7 Department of Economics and Statistics, Kabale University, Kabale, Uganda; City University of New York, UNITED STATES OF AMERICA

## Abstract

A substantial proportion (7%) of people in Uganda practice open defecation. A Community-Led Total Sanitation (CLTS) project was started in 2011 to reduce indiscriminate disposal of excreta but the effect has not been rigorously evaluated. We, therefore, evaluated the effect of CLTS on reducing open defecation in the program intervention districts in Uganda. We used the 2016 Uganda Demographic and Health Survey (UDHS) data to conduct a quasi-experimental study using a propensity score matching (PSM) approach. The intervention group consisted of districts that implemented the CLTS and the comparison group were districts that did not implement the CLTS. We matched the intervention and comparison groups on several covariates in a 1:1 ratio within a caliper of 20% of the standard deviation of the propensity score. We confirmed balance in covariates using standardized mean difference (SMD) being <0.1 or 10%. We applied a conditional logit regression model on the matched dataset adjusting for matched pairs to estimate the intervention effect, reported as odds ratio (OR) and 95% confidence interval (CI). We assessed the robustness of the estimate using Mantel-Haenszel sensitivity analysis. There were 17,415 participants (4,373 intervention vs. 13,042 comparison) before PSM and 8,470 participants (4,235 comparison vs. 4,235 intervention) after PSM. In the unmatched weighted dataset, 5.4% of participants had open defecation (7.9% intervention vs. 4.6% comparison). In the matched weighted data, open defecation was 9.3% and 7.6% in the comparison and intervention groups, respectively. Results show that CLTS reduced the prevalence of open defecation by 37% (OR 0.63, 95% CI 0-54-0.74) and this finding is robust to unmeasured confounders (Gamma = 1.35, p = 0.052). We recommend the scale-up of CLTS to the remaining districts by the Government of Uganda to reduce open defecation.

## Introduction

Access to adequate sanitation is a fundamental human right that promotes good health, well-being, and dignity but is a key challenge in most developing countries. Globally, 3.6 billion people lack access to sanitation services, with 673 million practicing open defecation [1]. In Sub-saharan Africa, 737 million people lack access to basic sanitation services, with 197 million practicing open defecation [2]. About 7% of the households in Uganda practice open defecation which negatively impacts human health by increasing the risk of diarrheal diseases, especially among children below 5 years of age [3–5].

Diarrheal diseases can be prevented through adequate sanitation, hygienic practices, and drinking safe water [6]. The Republic of Uganda and its partners have implemented several approaches to support behavioral change for improved sanitation and hygiene such as participatory hygiene and sanitation transformation and home improvement campaigns. In 2015, the Uganda Ministry of Health launched a community-led total sanitation (CLTS) program to reduce open defecation in selected districts in Uganda [7], lasting for five years(2011–2015) with support from the Global Sanitation Fund. The CLTS approach in the intervention districts was implemented through a series of structured community mobilization activities, including sensitization meetings led by trained facilitators from the Ministry of Health, Uganda, and supported by the District Health Officers. The meetings aimed at raising awareness about the health risks associated with open defecation, supplemented by practical demonstration of contamination routes to motivate collective action [8]. Local leaders such as village chairpersons and community health workers also played a crucial role by leading meetings, conducting follow-ups, and monitoring progress toward open defecation-free (ODF) status. Although CLTS emphasizes no upfront financial subsidies for sanitation facility construction, financial and material support was provided in the form of facilitator training, hygiene monitoring tools, and visual aids. The intervention districts were the districts with a high prevalence of open defecation, recruited in a phased manner, followed by regular follow-up visits to maintain momentum, assessment of latrine construction [9], and the monitoring of behavior change. The program ended with ODF certification by the District Health Officers once the district reached a substantial level of ODF status. Aligned with the National Sanitation Policy and the Uganda Development Plan, Uganda aims to eliminate open defecation by 2025, with a specific focus on improving sanitation facilities and hygiene practices. Local governments often set their targets as part of this broader national initiative.

The approach focused on mobilizing the community to take collective action towards achieving open defecation-free status and bringing about wide-scale behavioral change that potentially leads to the abandonment of open defecation practices [1,3]. Although this approach has been proven to support and sustain sanitation practices [10–13]. In some communities, open defecation, particularly in the bushes or on the fields is seen as culturally acceptable and convenient, while in others communities view it as a private matter that should not be discussed or even addressed publicly. These cultural norms impact the success of sanitation programs like CLTS as they can create resistance to using latrines or changing the long-standing traditional and cultural practices of open defecation.

The implementation of Community-Led Total Sanitation (CLTS) in Uganda faced several challenges, including cultural resistance, logistical difficulties, and funding constraints. In some intervention districts, deep-rooted cultural beliefs around sanitation practices created resistance to adopting open defecation-free behaviors for example in Karamoja District [7]. Logistical challenges, particularly in remote and hard-to-reach areas, further hindered the consistent monitoring and support needed for sustained behavior change. Additionally, limited funding and resource allocation affected the capacity to scale up interventions and provide adequate sanitation facilities [9]. Despite these barriers, efforts continued to adapt the CLTS approach to local contexts and address the identified obstacles to ensure greater impact.

Previous research in Uganda has examined the impact of CLTS on sanitation and hygiene practices [14,15], but there remains a critical need for data specifically addressing its effect on open defecation to assess and track progress over time. Achieving Sustainable Development Goal 6, particularly target 6.2, which focuses on eliminating open defecation and ensuring universal access to adequate and equitable sanitation by 2030 [16], hinges on solid evidence of CLTS’s effectiveness. Despite the widespread implementation of CLTS, there is a lack of conclusive evidence to confirm whether its scale-up can yield the necessary results. Evaluating the impact of CLTS is essential for guiding Uganda’s Ministry of Health in its efforts to achieve an open defecation-free (ODF) status, as outlined in the 2021 National Accelerated Basic Sanitation and ODF Roadmap [15]. This study, therefore, evaluates the effect of CLTS on open defecation in Uganda, providing critical insights to inform national sanitation strategies and policy.

## Methods and materials

### Study setting and data source

Uganda is administratively divided into districts and by the time of the Uganda Demographic Health Survey (UDHS) of 2016, there were a total of 112 districts. For the UDHS survey, 15 regions were created to provide a representative sample. Each of the regions has several districts except for Kampala, the capital city of the country regarded as a separate region. Districts represent more localized areas where specific governance and administrative duties are carried out and this is where implementation of CLTS was undertaken.

This study used the 2016 Uganda Demographic Health Survey (UDHS) data. The 2016 UDHS sample comprised 20,910 households allocated across 15 regions based on a probability proportional to size approach. Within the 15 regions, sample allocation was conducted taking into account rural and urban locations. A two-stage sample selection approach was used at each Enumeration Area (EA) or cluster and the household level. In the first stage, 697 EAs were chosen with a probability proportional to the size of the regions. The second selection stage involved selecting a fixed number of 30 households per cluster. Overall, 19,588 residential households were successfully surveyed. For this study, we excluded data from Kampala and Karamoja districts for reasons. Kampala district has an extremely low prevalence of open defecation estimated at 0.2% [17] while Karamoja region has a higher prevalence of open defecation placed at 68% due to cultural beliefs on open defecation and nomadic life of the population in the area [18]. Including the data from these settings would have introduced substantial variation, potentially skewing the results and making it difficult to draw meaningful comparisons across the rest of the study regions. While exclusion helps mitigate the impact of these outliers, future research could explore alternative methods such as stratification or sensitivity analysis to address such variations without exclusion. Therefore, the data analyzed were for the 13 regions.

### Study design and study population

We used a quasi-experimental design by applying propensity score matching to mimic a randomized control trial (RCT). We identified districts within the six regions as intervention areas where CLTS was implemented, and districts within the remaining seven regions as a comparison where no CLTS was undertaken.

The Uganda MoH with support from the Global Sanitation Fund implemented the CLTS approach in the selected regions: Budaka, Butalejje in the Bukedi region; Bulambuli district in the Elgon region, Otuke, Kole, Lira, Dokolo, Apac, and Alebtong districts in Lango regions, and Arua, Koboko, Maracha, Yumbe, Moyo, Nebbi and Zombo districts in West-Nile region.

### Measurements

#### Intervention variable.

CLTS was the intervention of interest, measured on a binary scale—districts that participated in the CLTS formed the intervention group while those that did not participate in CLTS comprised the comparison group. CLTS was implemented in 15 districts in Uganda between 2011 and 2015.

#### Covariates of the study.

These covariates used for the generation of propensity scores were selected based on their relationship with the outcome, exposure, or both and thus correspond with the conditional independence assumption as recommended [19] and reported in an earlier study that used PSM [20]. In addition, we adopted the Risks, Attitudes, Norms, Abilities, and Self-regulation (RANAS) model of behaviour change in determining the variables to be used in generating the propensity scores. The RANAS model underscores the importance of psychosocial factors in determining households’ sanitation behaviours including open defecation practices. We considered psychosocial characteristics to include social and personal factors. We also used the conceptual frameworks on latrine utilization [21,22]. The social factors include location (rural/urban) and economic conditions (wealth quintile) while personal ones are framed by socio-demographic factors such as sex, age, education, number of children below 5 years, and marital status, and these variables were used in creating the propensity scores. Only the observable variables were selected as covariates since they were easy to match between the intervention and comparison groups.

### Study outcome

Open defecation was the primary outcome, measured as the proportion of households that used the bush for defecation.

### Statistical analysis

We used Stata Version 17 to analyze the data. Categorical data were summarised as percentages and frequencies. To reduce selection bias, we balanced covariates accounting for systematic differences between the intervention and comparison groups to resemble a randomized control trial. We conducted PSM analysis utilizing seven covariates that are known to influence the intervention from the literature but also based on biological and social understanding [21–23]. Using a logit model, we fitted the intervention as a function of the covariates, then predicted propensity scores based on the coefficients, and matched the groups on the propensity scores. For the matching, we used various matching strategies, including nearest neighbor matching with and without caliper adjustment, optimal pair, and optimal full matching. To avoid bias from far-off matches, a caliper calculated as 20% of the propensity score’s standard deviation was finally used in a 1:1 ratio. Using a back-to-back histogram, we evaluated the propensity score balance between the groups. We also statistically evaluated the covariate balance between the groups using absolute standardized mean difference (SMD), with an SMD < 0.1 considered indicative of a good covariate balance. Following a successful match, we employed a conditional binary logistic regression model with adjustments for the matched pairs to estimate the effect of the intervention (CLTS) on the primary outcome, reported as odds ratio (OR) and 95% confidence interval (CI).

### Sensitivity analysis and reporting of findings

We used the Mantel-Haenszel statistic to test for the robustness of the results to unmeasured confounders in PSM. As shown in previous studies that utilized PSM [24,25], a distant gamma value from the Mantel-Haenszel sensitivity analysis for the lower or upper bounds to change from a statistically significant value to a statistically insignificant value or vice-versa would suggest that the results are robust to unmeasured confounders.

### Ethical issues

The UDHS dataset is freely available to the public at https://dhsprogram.com/data/available-datasets.cfm. For this study, we requested the dataset from the DHS program (www.dhsprogram.com) and we were granted permission to analyze it. As DHS datasets are freely available to the public, no ethical approval was required for this study as reported in previous studies [24,26].

## Results

### Summary of study profile

We found 17,415 observations (13,042 comparison vs. 4,373 intervention) before PSM, which was reduced to 8,470 observations (4,235 comparison vs. 4,235 intervention) after PSM. In the unmatched weighted data, 971 (5%) participants had open defecation (8% intervention vs. 5% comparison). In the matched weighted data, the prevalence of open defecation was 9% (701/8,265), more in the comparison group (9%) than in the intervention group (8%). Table 1 shows the distribution of sociodemographic characteristics by the percentage of households practicing open defecation. Results before matching, show a higher percentage of open defecation in rural (8.1%), poorest (20.2%), and not educated (10.1%) households compared to those from urban (1.8%), richest (0.3%) and with a higher level of education (1.3%) respectively. After matching, rural, poorest and not educated households similarly show high differentials in open defecation compared to urban, richest, and those with higher levels of education.

**Table 1:** Unweighted Baseline participant characteristics before and after propensity-score matching.

	Before PS matching					After PS matching				
	Comparison	Intervention	Overall	Open defecation, n (%)		Comparison	Intervention	Overall	Open defecation, n (%)	
	(n = 13,042)	(n = 4,373)	(n = 17,415)	(n = 17,415)	SMD (%)	(n = 4235)	(n = 4235)	(n = 8,470)	(n = 8,470)	SMD (%)
Covariate	No. (%)	No. (%)	No. (%)			No. (%)	No. (%)	No. (%)		
Type of residence										
Urban	2,491 (19.1)	618 (14.1)	4469 (22.8)	55 (`1.8)		591 (14.0)	607 (14.3)	1,198 (14.1	33 (2.8)	−1
Rural	10,551 (80.9)	3,755 (85.9)	15119 (77.2)	1,158 (8.1)	31.4	3644 (86.0)	3,628 (85.7)	7272 (85.9)	819 (11.3)	
Sex of head of household										
Male	9,124 (70.0)	3,038(69.5)	13508 (69.0)	817 (6.7)		2992 (70.6)	2,973 (70.2)	5,965 (70.4)	569 (9.5)	
Female	3,918 (30.0)	1,335 (30.5)	6080 (31.0)	396 (7.5)	1.1	1,243 (29.4)	1,262 (29.8)	2,505 (29.6)	283 (11.3)	1
Age of head of household (years)										
≤19	261 (2.0)	49 (1.1)	343 (1.8)	19 (6.1)		40 (0.9)	46 (1.1)	86 (1.0)	7 (8.1)	
20-30	3,511 (26.9)	1,019 (23.3)	5226 (26.7)	354 (7.8)		1,000 (23.6)	1,010 (23.8)	2010 (23.7)	229 (11.4)	
31-40	3,324 (25.5)	1,110 (25.4)	4995 (25.5)	306 (6.9)		1,083 (25.6)	1,088 (25.7)	2171 (25.6)	210 (9.7)	
41-50	2,401 (18.4)	857 (19.6)	3580 (18.3)	194 (6.0)		825 (19.5)	815 (19.2)	1640 (19.4)	136 (8.3)	
51+	3,545(27.2)	1,338 (30.6)	5444 (27.8)	340 (7.0)	11.2	1,287 (30.4)	1,276 (30.1)	2563 (30.3)	270 (10.5)	−1.1
Wealth index										
Poorest	2,055 (15.8)	1,717(39.3)	4528 (23.1)	760 (20.2)		1,612 (38.1)	1,597 (37.7)	3209 (37.9)	652 (20.3)	
Poorer	2,802 (21.5)	1,083 (24.8)	3955 (20.2)	238 (6.1)		1,048 (24.7)	1,068 (25.2)	2116 (25.0)	131 (6.2)	
Middle	2,955 (22.7)	601 (13.7)	3602 (18.4)	129 (3.6)		607 (14.3)	599 (14.1)	1206 (14.2)	45 (3.7)	
Richer	2,919 (22.4)	594 (13.6)	3621 (18.4)	78 (2.2)	−58	588 (13.9)	593(14.0)	1181 (13.9)	23 (2.0)	0.2
Richest	2,311 (17.7)	378 (8.6)	3882 (19.8)	8 (0.3)		380 (9.0)	378 (8.9)	758 (8.9)	1 (0.1)	
Number of children 5 and under										
None	5,494 (42.1)	1,697(38.8)	8243 (42.1)	540 (7.5)		1.621 (38.3)	1,616 (38.2)	3,237 (38.2)	347 (10.7)	
1-4	7,511 (57.6)	2,660 (60.8)	11291 (57.6)	1,059 (9.4)		2,604 (61.5)	2,603 (61.5)	5,207 (61.5)	504 (9.7)	
5+	37 (0.3)	16 (0.4)	54 (0.3)	3 (5.7)	6.9	10 (0.2)	16 (0.4)	26 (0.3)	1 (1.9)	0.5
The highest education level attained										
No education	2,074 (15.9)	658 (15.0)	3436 (17.5)	276 (10.1)		623 (14.7)	638 (15.1)	1,261 (14.9)	188 (14.9)	
Primary	7,110 (54.5)	2,589 (59.2)	1018 (52.0)6	748 (7.7)		2,539 (60.0)	2,516 (59.4)	5,055 (59.7)	551 (11.0)	
Secondary	2,652 (20.3)	686 (15.7)	3926 (20.0)	167 (5.0)		692 (16.3)	670(15.8)	1,362 (16.1)	97 (7.1)	
Higher	1,206 (9.2)	440 (10.1)	2040 (10.4)	22 (1.3)	−2.6	381 (9.0)	411 (9.7)	792 (9.4)	16 (2.0)	0.7
Marital Status										
Never married	884 (6.8)	139 (3.2)	1260 (6.4)	53 (5.2)		128 (3.0)	131 (3.1)	259 (3.1)	13 (5.0)	
Married	9,009 (69.1)	3,250 (74.3)	13737 (70.3)	773 (6.3)		3,177 (75.0)	3,160 (74.6)	6,337 (74.8)	573 (9.0)	
Widowed	1,511 (11.6)	590 (13.5)	2309 (11.8)	193 (9.2)		538 (12.7)	566 (13.4)	1,104 (13.0)	149 (13.5)	
Divorced	1,638 (12.6)	394 (9.0)	2243 (11.5)	194 (9.6)	−3.2	392 (9.3)	378 (8.9)	770 (9.1)	117 (15.2)	0.2

**Note: SMD < 10% either before or after propensity-score matching suggests covariate balance.**

### Covariate balance before and after PSM

Table 1 shows the covariate distribution before and after PSM. Before PSM, we found an imbalance in several covariates, namely type of place of residence, age of head of household, and wealth index (all SMD > 10%). However, after matching, the results show that all covariates were balanced between the intervention (n = 4,235) and comparison groups (n = 4,235).

### Additional balance diagnostics

**Fig 1:**
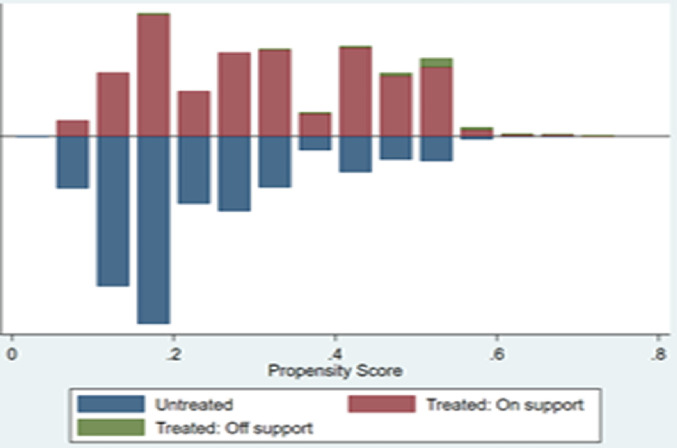
Distribution of propensity scores between the intervention and comparison groups before matching.

**Fig 2:**
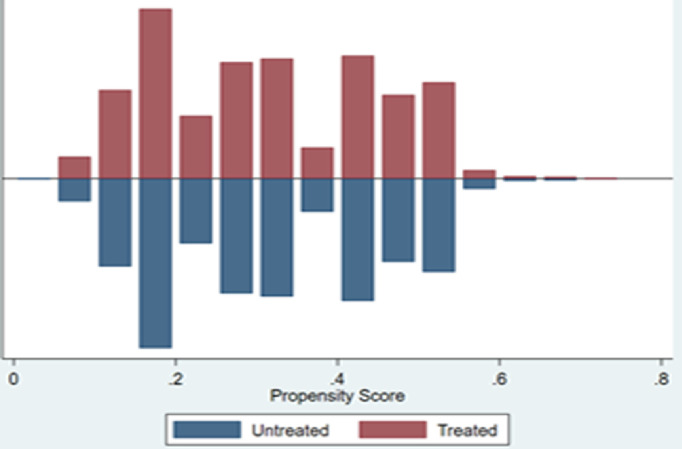
Distribution of propensity scores between the intervention and comparison groups after matching.

Figs 1 and 2 depict the distribution of propensity scores among the intervention and comparison participants. Before PSM (Fig 1), some of the propensity scores were off the region of common support (unmatched). However, after PSM (Fig 2), all the propensity scores were in the region of common support.

### Impact of CLTS on open defecation in 15 districts in Uganda between 2011 and 2015

Table 2 shows the weighted outcome both before and after PSM. Before PSM, open defecation was 8% (322/4,077) in the intervention group vs. 5% (649/14,052) in the comparison group (OR 1.77, 95% CI 1.54–2.04). After PSM, open defecation was 8% (301/3,955) in the intervention group compared to 9% (401/4,310) in the comparison group. In a PSM analysis, the intervention group had a 37% reduced likelihood of open defecation (OR 0.63, 95% CI 0.54–0.74).

**Table 2:** Impact of CLTS on open defecation (weighted) in 15 districts in Uganda between 2011 and 2015.

Outcome	Level	Before PSM		After PSM		PSM analysis
		Intervention	Comparison	Intervention	Comparison	
		No. (%)	No. (%)	No. (%)	No. (%)	OR (95% CI)
Open defecation	No	3,755 (92.1)	13,403 (95.4)	3,654 (92.4)	3,909 (90.7)	1
	Yes	322(7.9)	649(4.6)	301 (7.6%)	401 (9.3)	0.63*** (0.54–0.74)

Note: *** p < 0.001.

### Sensitivity analysis results

Using the Mantel-Haenszel sensitivity analysis, we determined that the gamma value shifted from a statistically significant to a statistically non-significant upper bound at 1.35 (p = 0.052), which was far from the point of no hidden bias (Gamma value = 1.0). The shift meant that for the current results not to hold as a result of unmeasured confounders, it had to change 135 times, which is a larger change from the point of no change. The gamma value, therefore, suggested that the findings are robust to the analytical method and unmeasured confounders.

## Discussion

We evaluated the effect of CTLS on open-free defecation in Uganda using the 2016 UDHS data. CLTS is a participatory approach in which the local people improve their sanitation practices to promote a sanitation-free environment. Our results show that 7% of the households in Uganda practice open defecation and there was a 37% reduction in open defecation as a result of CTLS. Studies carried out in Kenya and Ethiopia also found a significant role played by CLTS in increasing latrine ownership and reducing open defecation [27,28]. In Mali, access to a private latrine almost doubled among households in CLTS villages (65% in CLTS villages vs. 35% in control villages) following a CTLS program [29]. Similarly, a study carried out in urban areas of Tamale Metropolis found that CLTS had significantly reduced open defecation and recommended a scale-up in other communities where the practice was rampant. A study done in only one district (Pallisa district) in eastern Uganda [15] found that the knowledge of sanitation and hygiene significantly improved amongst households in CTLS intervention areas by about 10 percentage points compared to households in a non-CLTS intervention area. Also, CLTS intervention areas tended to have cleaner pit latrines compared to the non-CLTS intervention sites. Therefore, our results collaborate well with findings from several studies that show CLTS has a positive impact on reducing open defecation.

Our results also show a higher percentage of open defecation in rural, poorest, and not educated households compared to those from urban, richest, and with higher levels of education. Socio-demographic characteristics are associated with open defecation. A study conducted in Haiti [30] reported that to eliminate open defecation by 2030, there is a need for the government and its partners to consider wealth disparities among its regions (rural and urban). A study [31] that analyzed DHS datasets from 33 countries in sub-Saharan Africa found that open defecation was associated with educational attainment, household wealth status, and residence (rural/urban), similar to our findings.

The findings that CLTS reduces open defecation suggest a need for the Government of the Republic of Uganda and its partners to scale up the programme to other districts that are not implementing CLTS. Our findings also have implications for childhood diarrhoea. Open defecation contaminates the environment, leading to fecal-oral transmission of pathogens especially when flies carry pathogens from feces to food and surfaces. Evidence shows that open defecation increases diarrheal disease risk by 25% and achieving open defecation free (ODF) has been shown to reduce diarrheal disease incidence in children by 55%. In children, achieving ODF status interrupts and stops this fecal-oral transmission route, hence reducing diarrheal disease morbidity and mortality.

However, there are potential challenges in CLTS implementation [32]. For example, CLTS operates on the premise of behavioral transformation at the grassroots level, with a primary focus on ending open defecation through collective community efforts. The approach leverages shame, disgust, and pride as emotional triggers to inspire change, helping them recognize the adverse health effects of open defecation. Sometimes, few people have been opposed to this approach. The participatory nature of the approach, where the community members themselves take charge of constructing latrines and maintaining sanitation standards, fosters a sense of ownership and pride. Evidence from field studies and community feedback indicates that visual and emotional appeals were especially effective in motivating individuals to adopt safer sanitation practices. However, success varied depending on the level of community cohesion and the presence of local champions who could drive and sustain momentum.

Challenges remain, especially in achieving sustained behavior change. In some cases, the initial enthusiasm wanes over time, and without continuous reinforcement, some communities revert to old practices. The presence of community members who spearheaded the initiative was critical in ensuring long-term success and preventing relapse [8,33]. The CLTS program in Uganda tackled these cultural barriers through a combination of community discussions, education, and demonstrations. Facilitators encouraged open conversations about defecation practices, which are often taboo subjects, and linked the idea of sanitation to improved health outcomes, using culturally relevant examples. For instance, in some areas, the construction of private household latrines was framed as a matter of dignity and social standing, which resonated strongly with local customs surrounding cleanliness and family honor.

Achieving cultural acceptance of latrines remains an ongoing challenge in certain areas. In communities where traditional practices of open defecation are particularly strong, resistance to change was observed, despite the awareness of the health risks associated with open defecation. A study in northern Uganda found that while knowledge of the benefits of latrines was high, actual usage rates remained low due to cultural resistance [17,34]. The study suggested that greater involvement of cultural and religious leaders, who hold significant influence in these communities, could help mitigate these challenges by endorsing latrine use as compatible with traditional values. In some regions, CLTS also promoted alternative sanitation solutions, such as eco-friendly latrines to address cultural concerns about waste handling. These innovations helped to alleviate some of the discomforts that community members had about conventional latrines, thus improving the overall uptake of sanitation practices. However, more targeted interventions that address specific cultural concerns may still be required for full cultural integration of CLTS [17].

### Implications of findings for policy, practice, and research

The scalability and sustainability of CLTS across Uganda require a multifaceted strategy that addresses geographic, cultural, and economic diversity. Continuous training of facilitators and community leaders is crucial for adapting CLTS approaches to local contexts, achieved through expanding training programs and establishing regional hubs [35]. Engaging local leaders, including village chairpersons, and cultural and religious figures, in capacity-building and resource allocation can effectively promote the widespread adoption of sanitation practices within their communities [33]. Integrating CLTS into health and development programs, such as maternal health, nutrition, and education, can instill lasting hygiene behaviors in children, which can be transferred to households. A robust monitoring and evaluation framework is crucial for stakeholders to track progress, identify challenges, and adjust strategies, while also incorporating regular community feedback. Local committees or “sanitation champions” are crucial for sustaining change by engaging with community members, monitoring latrine usage, and reinforcing sanitation messages. Training on latrine maintenance and encouraging the development of community funds or micro-loans for latrine repairs and upgrades would enable long-term maintenance of latrines. CLTS may introduce incentives like awards for best-maintained latrines or recognition for ODF villages to motivate communities to maintain sanitation standards without external funding. Flood-prone regions might require eco-friendly or flood-resistant latrines to ensure long-term functionality and prevent unusability during rainy seasons.

CLTS is already aligned with the National Sanitation Policy and the Uganda Development Plan. Uganda aims to eliminate open defecation by 2025, with a specific focus on improving sanitation facilities and hygiene practices. However, a collaboration between the MoH, the Ministry of Education, and the Ministry of Water and Environment in integrating CLTS as part of their WASH programming is important. This integration would CLTS to receive consistent government support, funding, and oversight. Policy frameworks should empower local governments to take ownership of CLTS implementation, empowering them to create customized sanitation plans that cater to their unique cultural and environmental needs. Decentralization can improve efficiency and ensure that national sanitation policies are adapted to local realities [34]. Integrating CLTS principles into school curriculums, particularly in rural and peri-urban areas, can foster a generation that values sanitation and hygiene, complemented by school-based WASH programs. The private sector can significantly enhance CLTS by constructing latrines and supplying sanitation products, while government support and local entrepreneurs can encourage innovation for affordable sanitation solutions [33]. The Ugandan government should enhance collaboration with international organizations and NGOs for harmonized CLTS implementation, including clear policy frameworks, joint funding mechanisms, and shared monitoring frameworks.

To build on the findings of this study, future research should focus on longitudinal studies to assess the long-term effect of CLTS on reducing open defecation and improving sanitation behaviors, as well as its effect on other health outcomes like diarrheal diseases and stunting in children. Research on the cost-effectiveness of CLTS is crucial for comparing it with other sanitation interventions, providing valuable evidence for policymakers and stakeholders. Future research should explore effective CLTS elements that drive the shift away from open defecation, behavior change triggers, and local leadership’s role in CLTS success, focusing on involving traditional leaders, women’s groups, and youth in sanitation advocacy.

### Study strengths

The study’s main strength includes reliance on nationally representative data hence providing credible evidence. We used a sound and methodologically robust analytic impact evaluation approach to provide a credible estimate of the intervention effect.

### Study limitations

The main limitations include several unmeasured confounders which would have improved the precision of the estimates had they been included. The absence of baseline data on sanitation practices and open defecation rates in both the intervention and comparison districts before the implementation of CLTS could not allow us to compare changes in the trends of ODF status. Longitudinal studies should be considered in the future to measure the long-term effect of CLTS on open defecation. However, sensitivity analysis showed that the finding is robust to unmeasured confounders. It is also likely that the study could have had spillover effects in the comparison districts hence underestimating the effect of CTLS on open defecation. We mitigated this problem by analyzing data from distant districts for the comparison group. Additionally, the study’s focus on certain districts may limit the generalizability of findings to some urban settings, where sanitation challenges and cultural contexts differ significantly. Furthermore, the long-term sustainability of behavior change could not be assessed within the study period since we used a non-experimental method where follow-up data collection was not employed. Finally, the lack of qualitative data to contextualize the findings should be considered.

## Conclusions and recommendations

We found that CLTS led to a reduction in open defecation in intervention districts compared to the comparison districts in Uganda. There is, therefore, a need for the Government of Uganda to expand the CLTS to non-intervention districts in order to reduce the prevalence of diarrhoeal diseases including diarrhea-related mortality.

## Supporting information

S1 File List of regions by CLTS intervention and comparison sites. (DOCX)
