# Supplementary material for: Effect of community-led total sanitation on open defecation in Uganda: A propensity score-matched analysis
Source: PLoS One. 2025 Jul 24;20(7):e0329307. doi: 10.1371/journal.pone.0329307 (PMC12288991; doi:10.1371/journal.pone.0329307)
Supplement: S1 File — (DOCX) [file pone.0329307.s001.docx]

| **No** | **Sub-regions** | **Districts (2011-2015)** | **Districts (2014-2015)** | **Intervention** |
| --- | --- | --- | --- | --- |
| 1 | Bunyoro | No | No | No |
| 2 | Busoga | No | No | No |
| 3 | Acholi | No | No | No |
| 4 | Buganda North | No | No | No |
| 5 | Buganda South | No | No | No |
| 6 | Kigezi | No | No | No |
| 7 | Tooro | No | No | No |
| 8 | Ankole | Bushenyi, Sheema & Mbarara |  | Yes |
| 9 | Bukedi | Pallisa, Kibuku | Budaka, Butalejja | Yes |
| 10 | Elgon |  | Bulambuli | Yes |
| 11 | Lango | Amolator | Otuke, Kole, Lira, Dokolo, Apac, Alebtong | Yes |
| 12 | Teso | Kaberamaido, Serere, Bukedea, Kumi, Ngora, Soroti, Amuria, Katakwi |  | Yes |
| 13 | West-Nile |  | Arua, Koboko, Maracha, Yumbe, Moyo, Nebbi, Zombo | Yes |
| 14 | Kampala |  | No | Excluded |
| 15 | Karamoja |  | No | Excluded |

S1 File. List of regions by CLTS intervention and Comparison
